# Successful response in advanced leptomeningeal disease from pleomorphic xanthoastrocytoma with BRAF/MEK inhibitors: a case report

**DOI:** 10.3389/fonc.2025.1669942

**Published:** 2025-11-21

**Authors:** Maria A. Jacome, Robert J. Macaulay, Edwin Peguero, Sheethal Cyriac, Solmaz Sahebjam, Arnold B. Etame, Hsiang-Hsuan Michael Yu, Yolanda Piña

**Affiliations:** 1 Department of Immunology, H. Lee Moffitt Cancer Center & Research Institute, Tampa, FL, United States; 2 Department of Anatomic Pathology, H. Lee Moffitt Cancer Center & Research Institute, Tampa, FL, United States; 3 Department of Neuro-Oncology, H. Lee Moffitt Cancer Center & Research Institute, Tampa, FL, United States; 4 Department of Radiation Oncology, H. Lee Moffitt Cancer Center & Research Institute, Tampa, FL, United States

**Keywords:** leptomeningeal disease, pleomorphic xanthoastrocytoma, BRAF V600E mutation, BRAF inhibition, MEK inhibition, CNS tumor

## Abstract

Leptomeningeal disease (LMD) is a rare metastatic complication with a grim prognosis for most patients and limited treatment strategies. Therapy is adjusted to the primary tumor from which it arises. Targeted therapies and personalized medicine have become cornerstones in cancer treatment but its utility in LMD has been limited. In here we report a case of a female patient who developed LMD from a Pleomorphic Xanthoastrocytoma (PXA), BRAFV600-mutated, who has shown successful response to treatment with BRAF/MEKi (Encorafinib/Binimetinib) for over 3 years since initial LMD diagnosis. The effectiveness of therapy in this patient was initially observed as stable disease, with radiographic progression when BRAF/MEKi were withheld, and immediate tumor control achieved when reinstated. Despite being just one case, this hopefully could serve as proof-of-concept for use of targeted therapy for BRAF V600E-mutated tumors with LMD progression, sparing patients from alternative tumor control options such as radiation therapy.

## 1 Introduction

Leptomeningeal disease (LMD) is a rare complication of cancer arising from the spread of tumor cells to the leptomeninges and the cerebrospinal fluid (CSF) (1). LMD usually portrays a grim prognosis of only weeks to a few months depending on the subjacent primary tumor and the available standard-of-care therapies (2–4). Most common etiologies include melanoma, lung, and breast cancer, with primary brain tumors being less common and nearly no available data about diagnosis, development, and treatments (5–7).

Here, we report a case of a patient with a longstanding history of a primary brain tumor, a Pleomorphic Xanthoastrocytoma (PXA), BRAF V600E mutated WHO grade 2-3, who developed CSF spread and has been outstandingly controlled with a combination of B-raf (BRAF) and Mitogen-activated protein kinase (MEK) inhibitors (BRAF/MEKi) for over 3 years.

## 2 Case presentation

**Figure 1:**
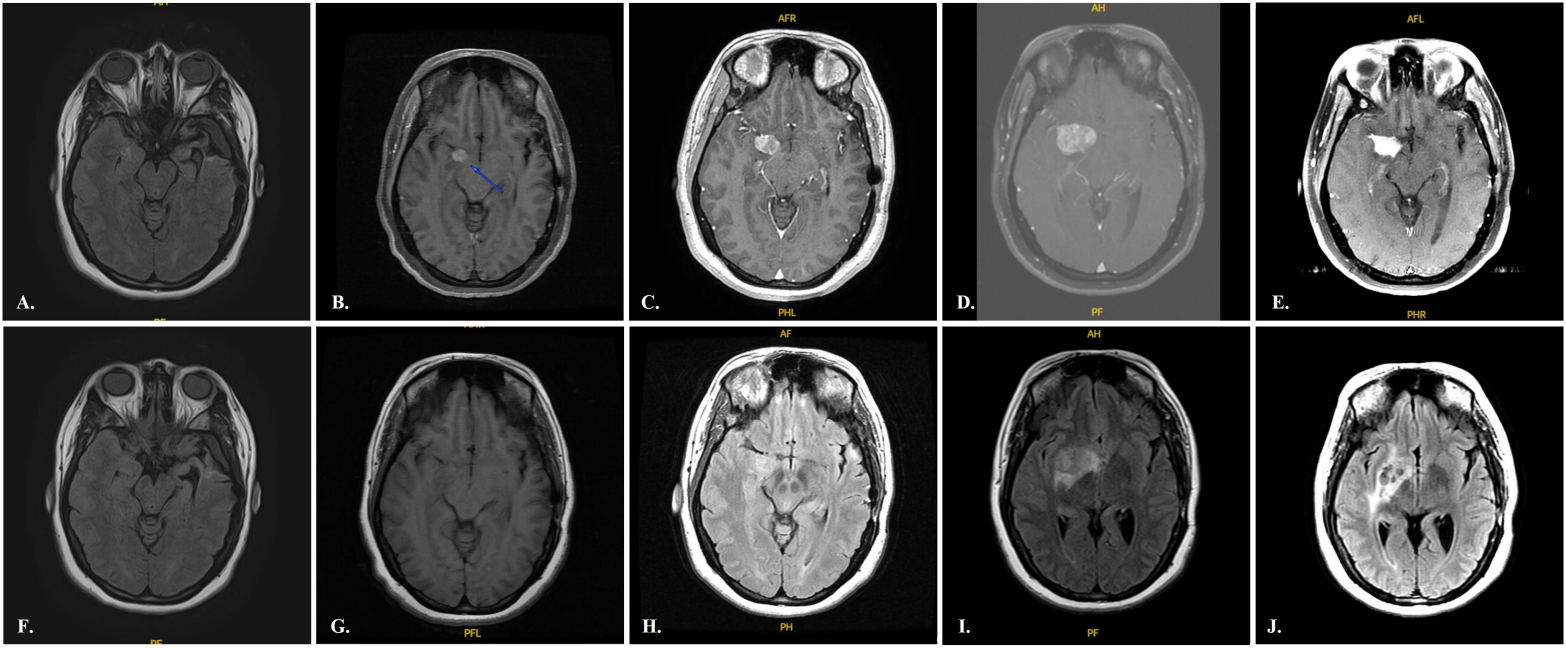
MRI of the brain showing stable post-surgical changes after first tumor resection, while on surveillance (**A, F**, 01/13/2014). New enhancing lesion develops in the medial right temporal lobe 7 x 11 x 12 mm (**B, G**, 03/09/2015), increasing in size and in surrounding vasogenic edema (**C, H**, 08/19/2015) and significantly continues to increase in size (**D, I,** 02/29/2016). Chemoradiation is started followed by maintenance cyclic temozolomide and the tumor shows shrinkage after 12 cycles of temozolomide (**E, J**, 09/26/2017).

In September 2015, a 31-year-old female originally from Puerto Rico presented to our Neuro-Oncology clinic at Moffitt Cancer Center (MCC), in Tampa, Florida, looking for a second opinion regarding a lesion found in brain Magnetic Resonance Imaging (MRI) and worsening of her long-standing seizures. The patient had a past medical history of obesity, asthma, and generalized tonic-clonic (GTC) seizures that began at the age of 19. At 23 years old, she had an MRI of the brain completed due to increased severity and frequency of her seizures despite multiple antiseizure drugs. MRI brain revealed a left temporal lobe lesion (images not available). She underwent a left temporal craniotomy in Puerto Rico on 12/13/2007. Pathology reported a Pilocytic astrocytoma WHO grade 1, with immunohistochemistry reporting a strongly positive Glial Fibrillary Acidic Protein (GFAP), a low Ki-67, and Neu N positive. She did not receive chemotherapy or radiation therapy at the time (access to this tissue is unavailable to us). Seizures subsequently improved and were controlled with only Levetiracetam (Keppra). She remained radiographically stable until 01/13/2014 (**Figures 1A, F**), when she had recurrence of GTCs. MRI brain in 03/09/2015 (**Figures 1B, G**) showed a new enhancing lesion in the medial right temporal lobe measuring 7 x 11 x 12 mm. This lesion continued to increase in size showing surrounding vasogenic edema (**Figures 1C, D, H, I**). Treatment is initiated with chemoradiation (5940 cGy in 33 fractions to the right temporal region) with concurrent temozolomide (TMZ) at 75 mg/m2 which she completed on 05/11/2016, followed by 12 cycles of maintenance TMZ at 150–200 mg/m2 once a day for 5 days in a 28-day cycle, with successful shrinkage of the enhancing lesion (**Figures 1E, J**).

Patient remained radiographically stable for more than 2 years, until MRI brain showed a new enhancing lesion on T1 in the left middle cranial fossa where her first resection was completed on 05/08/2019 (images not shown). She was re-challenged with maintenance cyclic TMZ again 150–200 mg/m2/day for a total of 11 cycles, with a good response to treatment radiographically and clinically, and seizures controlled with Levetiracetam 1500mg PO twice a day (BID). The patient developed new onset headaches five months after completing the last dose of TMZ and she was started on Topiramate 50mg PO BID with mild relief.

**Figure 2:**
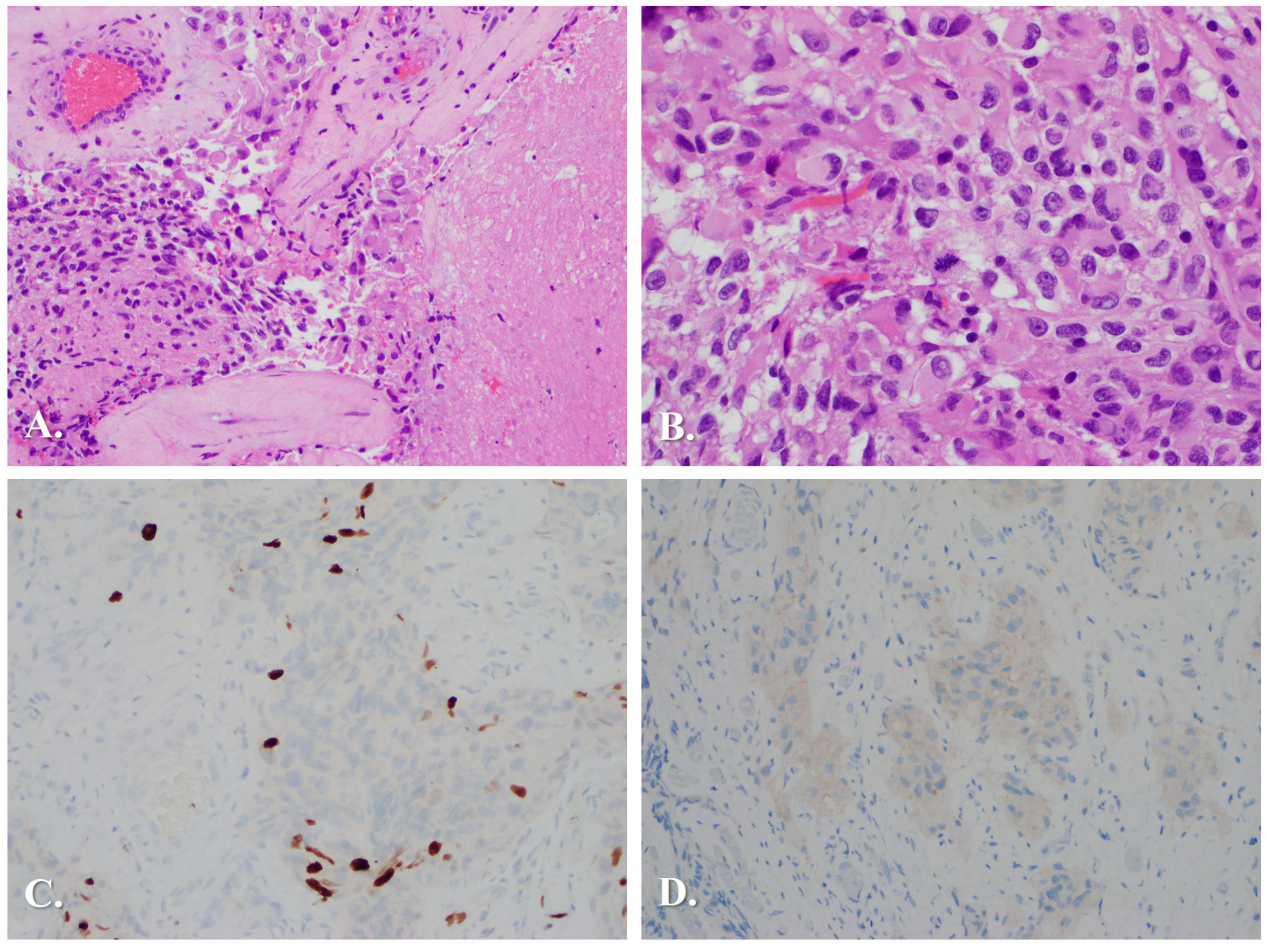
Histopathology of tumor samples from 2021 left temporal resection. **(A)** Vascular proliferation associated with hyalinization and necrosis, attributed to treatment effects. H&E, x200. **(B)** Nuclear atypia, plump eosinophilic cytoplasm, fibrillary processes and scattered mitoses. H&E x400. Immunostaining showing: **(C)** Ki-67 proliferation index of 3%; and **(D)** Diffuse positivity for mutant BRAF V600E, both x200.

On 06/02/2021 (14 months after her last TMZ cycle), MRI brain showed growth of the enhancing lesion toward the mesial left temporal region from 1.5 x 1 cm to 1.9 x 1.5 cm. The scans also reported the previously stable right temporal lobe region had changes including a dural tail and associated multiple cysts which were suggestive of a pleomorphic xanthoastrocytoma (PXA) or a ganglioglioma. The patient was offered surgery, but she refused and instead was started on Lomustine (CCNU) 200mg PO once every six weeks. After 1 cycle of CCNU, the patient developed worsening symptoms of memory loss and episodes of metallic taste and smell. Subsequent imaging on 08/04/2021 demonstrated left-sided tumor size increased to 2.5 cm. She therefore underwent a left temporal stereotactic craniotomy with tractography for resection of the left-sided lesion. Pathology described vascular proliferation associated with hyalinization, patchy dense fibrosis, and focal necrosis. Neoplastic cells showed eosinophilic cytoplasm with fibrillary processes and oval hyperchromatic nuclei. Atypia and some mitoses were also seen (**Figures 2A, B**). Immunohistochemistry staining showed a Ki-67 proliferative index of 3% and faint positivity for BRAF V600E (**Figures 2C, D**). Some of these features favored the diagnosis of pleomorphic xanthoastrocytoma despite the absence of xanthoma cells or eosinophilic granular bodies. Foundation Medicine molecular testing was conducted (**Table 1**), demonstrating mutations of BRAF and PAX5, amplification of HGF, and CDKN2A/2B loss. Due to prior clinical history and the current progression with CDKN2A loss, interpretation supported the theory of the original tumor being a pleomorphic xanthoastrocytoma with novel anaplastic progression (WHO grade 3). Subsequent methylome profiling confirmed the diagnosis of PXA (DKFZ v12b6).

**Table 1:** Personalized medicine results.

BRAF V600E (mutation allele frequency 32.8%)
CDKN2B loss
CDKN2A p14ARF loss exon 1
HGF amplification
PAX5 H46fs*37 (mutation allele frequency 12.9%)
Tumor mutation burden: Low (3 mutations per megabase)
Microsatellite status: Stable
9 variants of unknown significance

**Figure 3:**
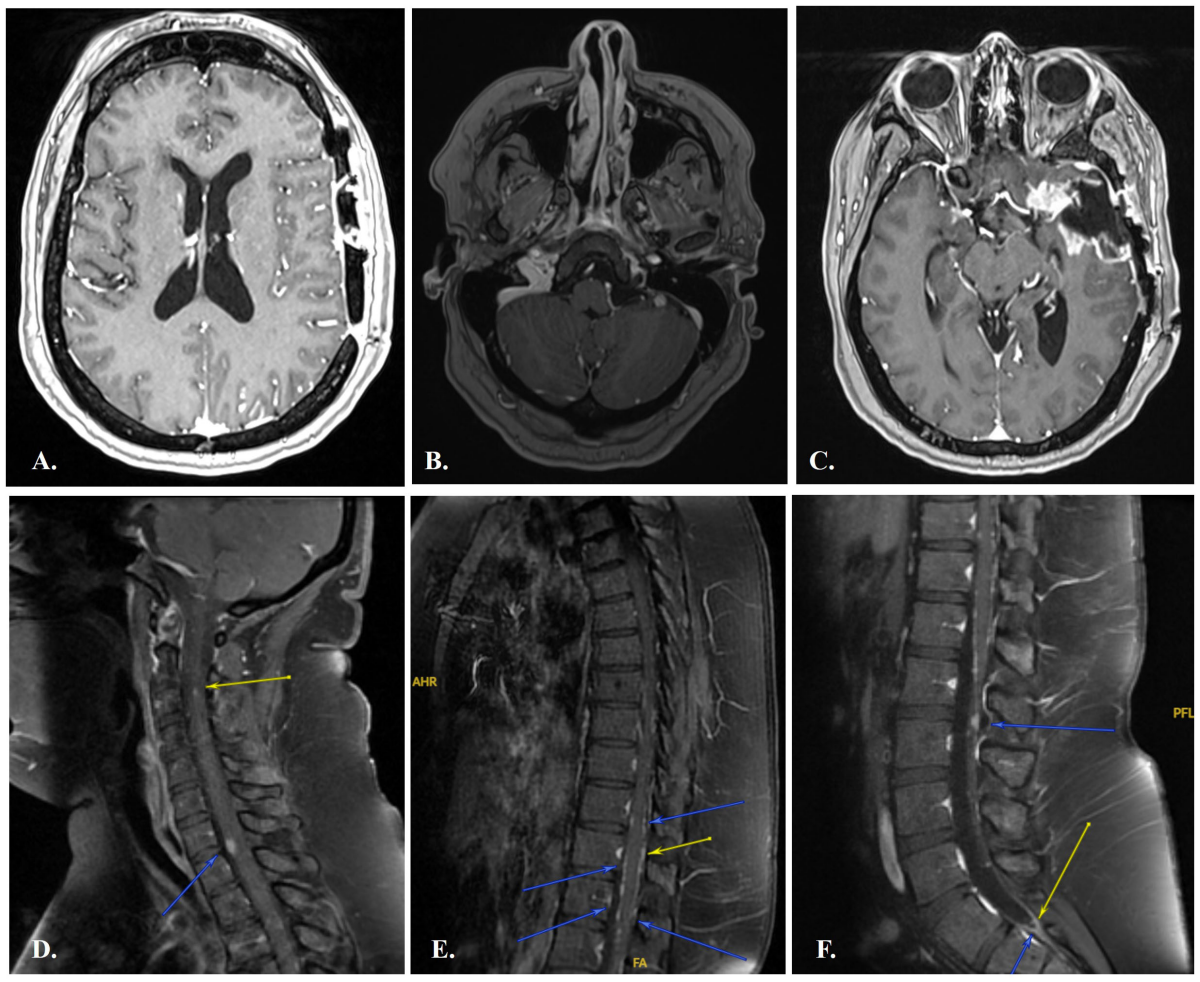
Leptomeningeal enhancement around the meninges, cerebellum and spinal cord at time of LMD diagnosis. **(A)**. axial T1 post-contrast sequences in which linear and nodular enhancement around the meninges can be seen, as well as curvilinear enhancement around the cerebellum **(B)**. **(C)**. shows post-surgical changes. A combination of linear and nodular enhancement categorizes this case as radiologic subtype C under the EANO–ESMO Clinical Practice Guidelines. Sagittal T1 post-contrast sequences of the spinal cord **(D–F)** with nodular leptomeningeal enhancement seen around the cervical spinal cord at the levels of C2-3 and C7-T1 **(D)**, along the mid thoracic cord **(E)**, and at the level of L2-3, the conus medullaris, and cauda equina, with enhancement also seen along the lower sacral cistern and thecal sac terminus **(F)**.

Plans were to re-challenge the recurrent disease in the left temporal region with the same regimen of concurrent chemoradiation therapy followed by adjuvant TMZ previously described or consider adjuvant BRAF/MEKi given her BRAF V600E mutation. However, in preparation for RT pre-treatment imaging on 10/01/2021 there was nodular leptomeningeal involvement in brain imaging (**Figures 3A, C**) and curvilinear areas of enhancement around the cerebellum (**Figure 3B**) concerning for CSF dissemination or leptomeningeal disease (LMD).

Upon work-up for LMD, MRI of the total spine on 11/04/2021 showed nodular leptomeningeal enhancement along the spinal cord (**Figures 3D–F**). T2-weighed FLAIR sequences showed no enhancement around the leptomeninges, ruling out vascular anomalies. A single lumbar puncture was performed, which showed an opening pressure of 19cm H_2_O. CSF analysis revealed a normal glucose concentration (55 mg/dL) with elevated total protein (88 mg/dL). Furthermore, myelin basic protein was high (11.10 ng/mL) which could be a marker of acute disease in LMD. The cellularity of the sample showed 18 nucleated cells with 60% monocytes and 39% lymphocytes, 8 red blood cells, and no malignant cells in cytology.

The rest of the CSF studies were unrevealing for infectious, inflammatory, or demyelinating etiologies. Despite negative cytology results, the argument for LMD was strong since a single sample of CSF can have a sensitivity as low as 50-60% if cells counts are low (8, 9). Repeating sampling can increase sensitivity and was recommended, but the patient refused to complete a second LP at the time.

In the presence of radiographic evidence of LMD with classical clinical signs such as worsening headaches, this case classifies as a probable LMD, type II, radiologic subtype C, with nodular and linear features on MRI, according to the EANO–ESMO Clinical Practice Guidelines (1).

**Figure 4:**
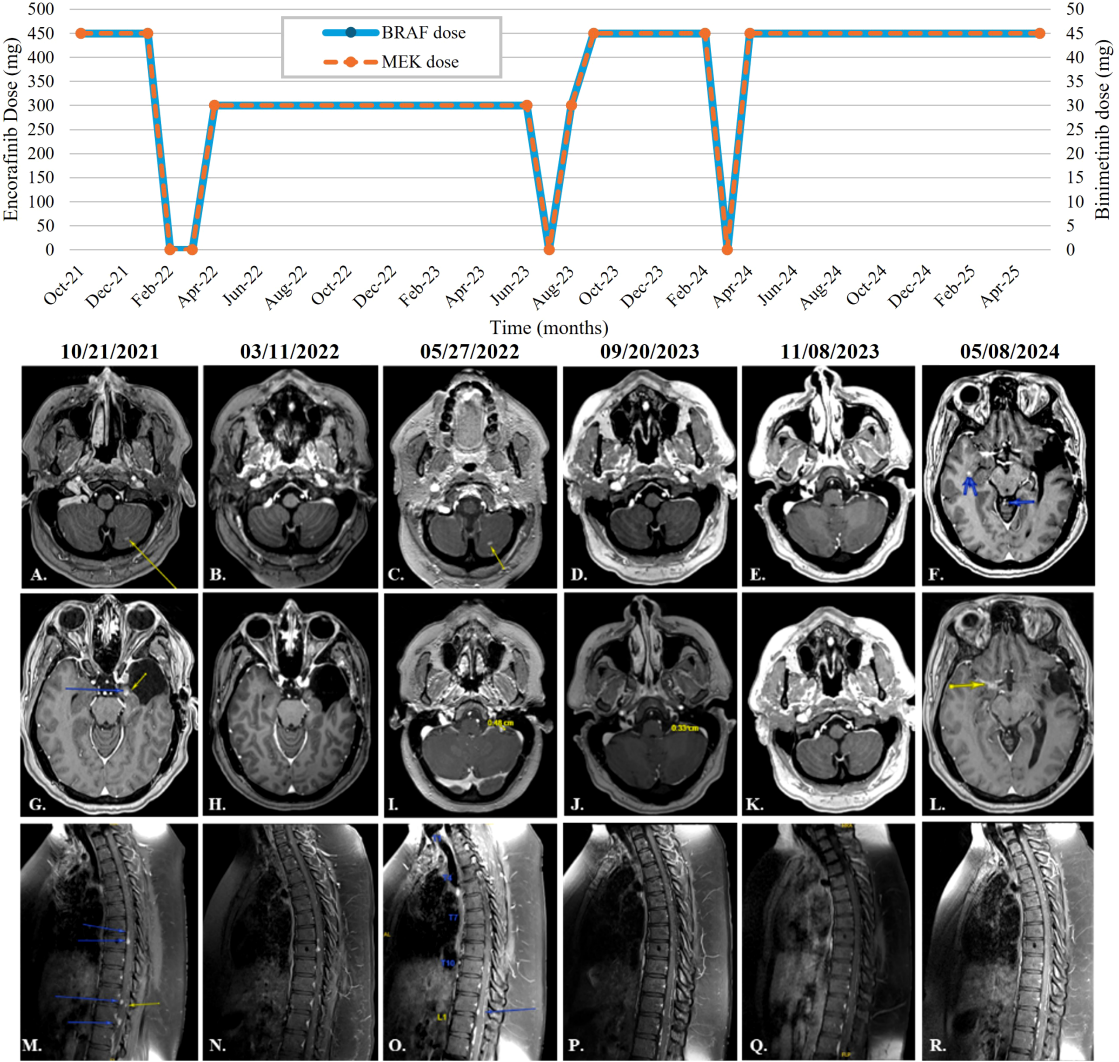
BRAF / MEK inhibitors dose through time and patient response. **(A, G, M)** show patient's baseline at time of LMD diagnosis and starting BRAF/MEKi therapy **(B, H, N)** show progression after therapy was held for four weeks. **(C, I, O)** show radiographic control and size reduction of enhancing nodular areas. **(D, J, P)** show new areas of enhancement after three days withholding the reduction dose of BRAF/MEKi. **(E, K, Q)** show radiographic improvement again after restarting full dose of BRAF/MEKi. **(F, L, R)** show new nodular enhancement in several areas of the CNS including a new temporal lesion after 2 weeks withholding BRAF/MEKi.

The patient started BRAF/MEKi therapy without radiation therapy. On 10/25/2021, she received Encorafinib 450mg PO daily and Binimetinib 45mg PO twice a day. Images at the time were considered patient’s baseline (**Figures 4A, G, M**). Our group had previously seen good responses in a small group of patients with V600E mutated gliomas, including a patient with PXA with the use of BRAF/MEKi (10, 11) which served as proof-of-concept to pursue this therapy.

The patient’s response to BRAF/MEKi therapy was immediate and the disease remained stable. However, after six months of therapy, the treatment was withheld for four weeks due to heavy vaginal bleeding. This led to the progression of her LMD in both the brain and the spine (**Figures 4B, H, N**). The patient restarted BRAF/MEKi therapy on a first reduction dose with Encorafinib 300mg PO once daily and Binimetinib 30mg PO BID, leading to direct radiographic control and size reduction of the enhancing nodular areas (**Figures 4C, I, O**). Vaginal bleeding resolved, as well as other side effects due to therapy such as grade 1 rash in the face and axilla.

Due to increased headaches the patient agreed to a second LP in April 2023. Results this time showed normal glucose and protein concentration, no nucleated cells, 1 red blood cell, and no malignant cells present.

The patient developed new onset weakness of the left eyebrow and was admitted for three days at an outside hospital where BRAF/MEKi therapy was once again withheld. Subsequently, her next MRI control showed new areas of enhancement (**Figures 4D, J, P**). It was decided to increase both drugs to full dose again and radiographic control and symptom improvement ensued (**Figures 4E, K, Q**). In March 2024, she was found to have moderate anemia (8.4 mg/dL) after an episode of rectal bleeding. BRAF/MEKi therapy was withheld for two more weeks until her anemia improved. The patient was also admitted to an outside facility for surgery due to gallstones. During this time, she was also diagnosed with gastritis and diverticulosis. When she returned to clinic, MRI brain showed a new temporal lesion (**Figures 4F, L, R**). This strengthened the reasoning supporting the direct role of BRAF/MEKi targeted therapy for disease control in this case.

**Figure 5:**
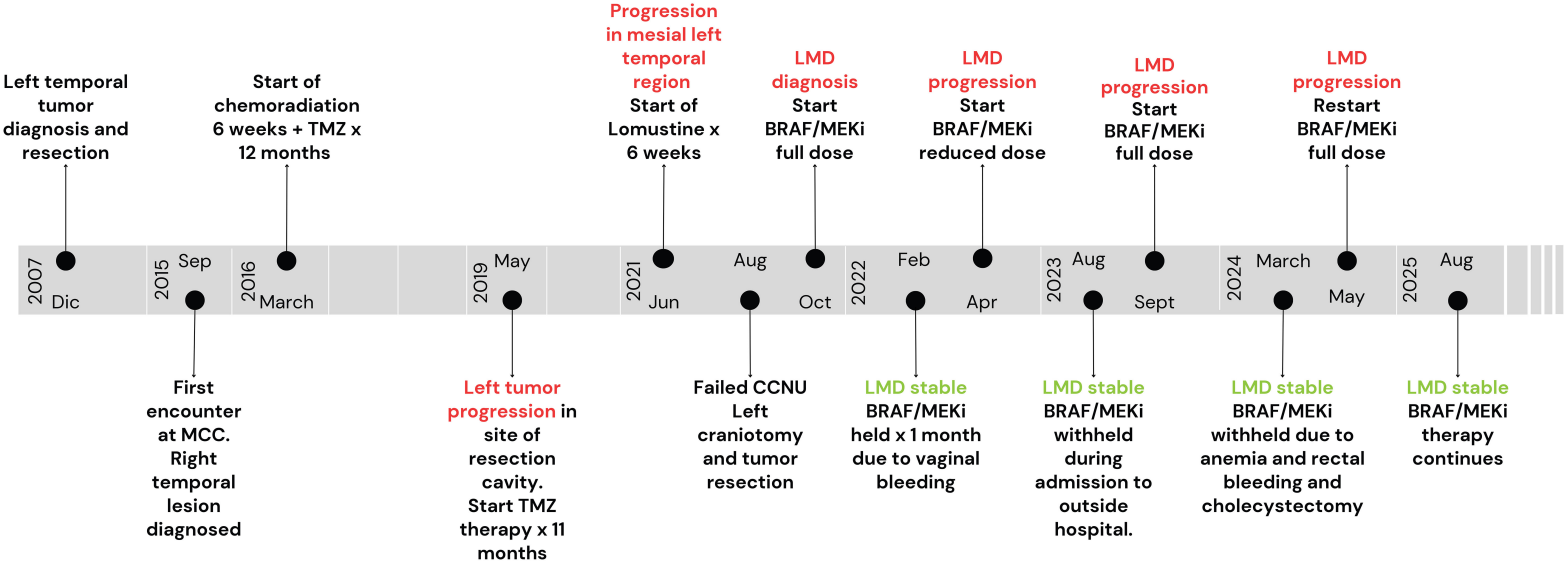
Complete case timeline since original tumor diagnosis. Tumor progression is highlighted in red, therapeutic response/stability to BRAF/MEK inhibitors is highlighted in green.

Overall, patient tolerance to this regimen has been acceptable with only grade 1 and grade 2 adverse effects to medication as discussed above and addressed accordingly together with the appropriate specialists. More recently she has also started to develop spurs in her hands and feet which remains to be ascertained as a side effect of BRAF/MEKi. The patient continues to have optimal seizure control with Levetiracetam 1500mg PO BID and headache control with Topiramate 50mg PO BID. Her Karnofsky performance score (KPS) has remained 90% from the neurological standpoint. The patient is oriented to person, place, date, and situation. Speech and comprehension are intact and there is only a slight short memory impairment. Neurological examination is otherwise within normal limits. A complete timeline of the case can be seen in **Figure 5**.

## 3 Discussion

PXAs are rare, central nervous system (CNS) tumors, as they represent less than 0.1% of newly diagnosed CNS tumors (12). The 2021 WHO Classification of Tumors of the CNS classifies PXAs under the umbrella of circumscribed astrocytic gliomas, with large and frequently multinucleated cells, surrounded by a reticulin network, and numerous eosinophilic granular bodies (13). Since the 2016 update of the WHO classification of CNS tumors it can be categorized as either grade 2 or grade 3, if the mitotic index is equal or greater than 5 mitoses per 10 high powered fields (HPF) (13–15). However, grading of PXA remains controversial. PXA grade 3 was previously known as anaplastic PXA (15).

Patients usually present with headaches and seizures (16). Radiographically, it could appear as a solid mass with cystic changes or a cystic mass with a mural nodule or uneven wall thickness, commonly located in the temporal lobe (17). These characteristics overlap with some malignant gliomas or glioblastomas, making misdiagnosis common. Infiltrative margins can also be seen and attachment to the meninges or the lateral ventricles occurs in over half of all the PXA cases (17–19). Leptomeningeal spread from PXA tumors has been described before in both children and adults (20–28). Zagardo et al. recently published a case of a patient with LMD from a BRAF V6000E mutated PXA in which spinal RT was offered on top of BRAF/MEKi (29). We believe this is the first case reported of an adult with PXA and LMD in which only BRAF/MEKi targeted therapy has been used for disease control.

The RAF family proteins are serine/threonine protein kinases involved in the mitogen-activated protein kinase (MAPK) pathway, that can increase cell proliferation and survival when activated. There are three different RAF isoforms (A-RAF, B-RAF, and C-RAF), and they are activated by RAS proteins upstream in the MAPK pathway. Once activated, RAF proteins lead to downstream phosphorylation of MEK1 and MEK2 and subsequent downstream activation of ERK1 and ERK2 (30). However, mutations at any level of the pathway, such as BRAFV600 mutations, create a constitutively active BRAF kinase and unregulated pathway activation (31).

BRAF mutations are present in various gliomas, including gangliogliomas, pilocytic astrocytomas, PXA, and epithelioid glioblastomas (32–35). Original pathology from our patient’s resection in 2007 demonstrated a Pilocytic Astrocytoma, a low-grade tumor with generally favorable prognosis (36). This sample was not interrogated molecularly nor evaluated by our institution’s neuro pathologists and whether if it was a Pilocytic Astrocytoma that transformed to a PXA or if it was a molecular PXA all along are both valid arguments.

PXAs are characterized by frequent *BRAF* V600E mutations or *CDKN2A/B* deletions (37). BRAF V600E mutations are present in over 60% of PXA tumors (34, 37, 38). Having a BRAF mutation confers the advantage of being responsive to BRAF/MEKi (14). Whereas most data of the use of BRAF/MEKi comes from treatment of malignant melanoma tumors (39), there has been a widespread use of these targeted therapies in other primary cancer types holding this mutation such as non-small-cell lung cancer, anaplastic thyroid cancer, colorectal cancer, or primary brain tumors such as PXA (30). Small cohort series and single case reports have demonstrated success with this treatment in patients with gliomas (40–44). Our own group previously reported cases of PXA patients showing favorable responses to BRAF/MEKi (10, 11). However, to our knowledge, there are no reports of a PXA with LMD treated with BRAF/MEKi.

Since its first introduction in 2011, there have been multiple BRAF inhibitors being introduced, with variable responses achieved depending on the primary tumor and its location (30). Considering the brain and spine are shielded from the rest of the body by the blood-brain barrier (BBB), drugs for primary brain tumors must be selected based on effectiveness as well as their CNS penetrance. Vemurafenib, a selective inhibitor of BRAFV600, showed in clinical trials a good response in melanoma brain metastases (45). Later, the VE-BASKET trial included 7 patients with PXA being treated with vemurafenib, out of which one achieved complete response, two partial responses, and three achieved stable disease (44). Dabrafenib, a selective Raf kinase inhibitor, has also shown intracranial responses in metastatic BRAFV600 mutated melanoma (46). Combination of dabrafenib with the MEK inhibitor trametinib has also demonstrated favorable results in a specific case of PXA (43). Of note, this was a case of a patient with a primary PXA that progressed and developed skeletal, lung, cervical lymph node, and subcutaneous metastases.

For our patient we chose a combination of the BRAF inhibitor Encorafinib and the MEK inhibitor Binimetinib. In prior retrospective case series, this combination elicited intracranial response in melanoma brain metastases with BRAFV600 mutations (47). The combination proved also effective for melanoma brain and brainstem metastases that had been resistant to different prior BRAF/MEKi combinations (48, 49). Moreover, McLoughlin et al. reported a case of a BRAFV600-mutated NSCLC with LMD and brain metastases that showed improvement when initiated in Encorafinib plus Binimetinib (50) serving as evidence of the potential to reach the leptomeningeal compartment of this combination. Additionally, the safety profile observed with the combination of Encorafinib plus Binimetinib has showed a decreased burden of toxicity with long-term use when compared with other BRAF/MEKi combinations (51). Moreover, the patient was spared from further radiation therapy and its side effects.

To the best of our knowledge, this is the first case of an adult patient with LMD from a BRAF V600E-mutant PXA that has received a combination of BRAF/MEKi with satisfactory results. This case also highlights the importance of tissue collection and interrogation to guide management and broaden therapeutic options toward personalized medicine. An argument can be made to interrogate even lower grade gliomas such as Pilocytic Astrocytomas and provide early detection of BRAF mutations that could inform clinical and radiological monitoring.

This study has several limitations. As a case report, results from this study do not reflect the general population and cannot be generalized. Also, PXAs are very rare, and although we focused our attention on the BRAF V600E mutation, it is still undetermined if these results could serve a different tumor or patient population. Even though a prospective study is needed to accurately evaluate the use of targeted therapy in BRAF V600E mutant PXA, accrual may be limited due to its rarity. Therefore, we advise caution when deciding therapy and to account for individual patient’s situations.

## 4 Conclusion

This case study highlights the disease control that can be achieved with BRAF/MEKi in specific tumors in which there is a targetable mutation, as BRAF V600E. Moreover, the results obtained with this patient have been outstanding in the setting of such an aggressive entity as LMD. This confirms that 1) BRAF/MEKi can reach the leptomeningeal space in therapeutic concentrations and 2) despite heterogeneity of tumors, mutations such as BRAF V600E persist even when metastasizing to the leptomeningeal space and can be targetable. Additionally, successful results were achieved without the need to submit the patient to further radiation to the CNS.

## Data Availability

The original contributions presented in the study are included in the article/supplementary material. Further inquiries can be directed to the corresponding author.
